# A Novel Approach to Managing a COVID-19 Outbreak at a Farm in Outer Regional Victoria, Australia

**DOI:** 10.3390/nursrep12040071

**Published:** 2022-10-07

**Authors:** Mwila Kabwe, Jennifer L. Dittmer, Jaimee Oxford, Catina Eyres, Ancara Thomas, Andrew Mahony, Bruce Bolam

**Affiliations:** 1 The Loddon Mallee Public Health Unit, Bendigo Health, P.O. Box 126, Bendigo 3552, Australia; 2 Department of Rural Clinical Sciences, La Trobe Rural Health School, La Trobe University, P.O. Box 199, Bendigo 3552, Australia

**Keywords:** coronavirus disease (COVID-19), outbreak management, seasonal farmworkers, remote rural farms, culturally and linguistically diverse

## Abstract

The coronavirus disease (COVID-19) has been established as a major occupational health and safety issue that compounds pre-existing socioeconomic inequalities such as access to basic health services. This is exacerbated in migrant farmworkers who are an essential workforce in maintaining food supply across the country. An outbreak occurred in a remote part of Victoria with limited access to healthcare resources. Existing relationships allowed the Loddon Mallee Public Health Unit to quickly engage farm management and local pathology services and provide cultural and language support. After contact-tracing and comprehensive clinical review, rather than isolate positive cases, those who were asymptomatic and willing to work continued to do so whilst negative workers were in quarantine. Outbreak management and public health actions were quickly implemented even when the nationwide state-testing and contact-tracing systems were experiencing significant strain due to the rapid escalation in case numbers. Despite a large outbreak (68/74 workers), the management of the outbreak allowed asymptomatic cases to perform their work so farm productivity remained uninterrupted. Cases’ health status was closely monitored, with no adverse outcomes in a high-risk population. COVID-19 negative workers safely quarantined away from positive cases until the closure of the outbreak.

## 1. Introduction

Local farms are critical for maintaining food security in Australia and abroad [1,2]. In response to the global pandemic of the severe acute respiratory syndrome—2 (SARS-CoV-2) virus, the causative agent of the coronavirus disease (COVID-19), the Australian government and Department of Health (DH) highlighted guidelines for the management of COVID-19 across the country [3]. In farms specifically, the primary aim of these guidelines were to reduce or prevent the impact of COVID-19 on the health and productivity of farms through a COVIDSafe plan [4]. In Victoria, COVID-19 cases and outbreaks were ultimately managed by nine local public health units, including the Loddon Mallee Public Health Unit (LMPHU).

Not unique to Australia, seasonal farmworkers around the world are disproportionally affected by both communicable and non-communicable diseases [5]. In the COVID-19 pandemic, this was greatly exacerbated [6]. In Australia, foreign and temporary workers make up nearly 30% of the total workforce with a similar proportion among farmworkers [7]. When Australia shut its borders to international travel at the end of March 2020 [8], a shortage of farmworkers was quickly realised, emphasising the significance of imported labour from the Pacific Island nations through the Pacific Australia Labour Mobility (PALM) scheme, a partnership created in 2005 [9]. As COVID-19 vaccinations were mandated in Victoria in October 2021 for all workers, only the PALM scheme provided a fully vaccinated farm workforce protected from the COVID-19 severe disease [10].

In late December 2021 and early January 2022, in Colignan, a small outer regional town in northern Victoria, there was a significant COVID-19 outbreak among seasonal farmworkers. This is a region where the economy depends on the agricultural production of citrus, grapes, garlic, melons, asparagus, and almonds [11]. This major outbreak provided insights into the vulnerabilities and challenges faced in the implementation of public health control measures in a culturally and linguistically diverse (CALD) population. 

This report highlighted the epidemiological investigation and public health intervention approach aimed at limiting spread and morbidity whilst striving to maintain the productivity of the farm during a period where food security in Victoria was being impacted by rapidly rising numbers of COVID-19 cases.

## 2. Materials and Methods

### 2.1. Outbreak Investigation Team

This outbreak investigation and management was coordinated by the LMPHU, internally comprising a team of medical leads, communicable disease team leaders, public health officers, an epidemiologist, and an infection prevention control (IPC) consultant and support from the Department of Health Infection Prevention Control Advice and Response (IPCAR) team. To overcome hurdles in testing, PCR testing was outsourced to a local pathology provider and tertiary hospital, and linked into cultural support and language translation support services. Data collected were managed through the Victorian Government Department of Health Transmission and Response Epidemiology Victorian (TREVI) system [12]. 

### 2.2. Outbreak Detection and Case Finding

Notification of the outbreak was received by the LMPHU from the pathology provider with three confirmed cases identified; an assessment of the site was performed by public health officers in the LMPHU identifying an additional eleven symptomatic workers. Confirmed outbreak cases were epidemiologically linked workers with a positive COVID-19 test through either polymerase chain reaction (PCR; confirmed cases) or rapid antigen test (RAT; probable cases). Epidemiologically linked cases were cases who had contact with other previously confirmed cases for at least 15 min or 2 h cumulatively, within 48 h of either symptom onset or date of sample collection in asymptomatic cases [13]. Cultural and language translation services were employed to ensure speedy case confirmation and accurate information collection to facilitate contact-tracing. Infections were considered active within seven days of testing positive, as per DH case-definition guidelines [13].

### 2.3. Analysis

Case, contact, and site assessments were conducted over the telephone and via Microsoft Teams application. Data collected were securely stored and managed through the Victorian Department of Health TREVI system. Descriptive analysis of cases was performed in Microsoft^®^ Office Excel and R Studio using the EpiCurve package.

### 2.4. Public Health Interventions

Before the outbreak was identified, routine targeted vaccination programs run by the LMPHU vaccination outreach team meant that by the time the outbreak was declared on the 8 January 2022, all workers had received at least 2 doses of the Pfizer-BioNTech (COMIRNATY) vaccine. None of the workers were reported to have had their last dose of COVID-19 vaccination more than 6 months or less than 10 days before outbreak declaration (considered for vaccine protection efficacy) or had COVID-19 infection 6 weeks prior (considered re-infection window at the time of the outbreak) [13]. Immediately after receiving the outbreak notification, which occurred late on a Saturday afternoon, the farm was contacted by one of the LMPHU public health officers to undertake a risk assessment; this assessment identified that the seasonal workers lived onsite in accommodation that consisted of a single or double room, with shared facilities and communal spaces. The pathology provider was then contacted and requested to assist in performing onsite-testing for all workers; they delivered seventy rapid-antigen-testing kits the next day. Further testing was subsequently undertaken on the fourth and sixth days on all workers who had previously tested negative to COVID-19 to ensure that any late onset infections were ruled out. The cultural and language support services assisted in providing instructions for performing a RAT to all workers and also provided pastoral care support to all onsite. The IPCAR and IPC consultant liaised with the farm management to ensure that appropriate infection control measures were in place to limit infection transmission among workers. An outbreak management team (OMT) was established to discuss with all stakeholders and workers the optimal measures to manage the outbreak. During this meeting after consulting with the LMPHU medical leads, it was determined that all symptomatic workers would be treated as COVID-19-positive due to the delays in obtaining the results of COVID-19 PCRs that were occurring at the time. The local tertiary public hospital supported the outbreak by performing a clinical assessment on cases as they were identified; they then met with the site daily during the outbreak to monitor the clinical status of all cases.

## 3. Results

### 3.1. Epidemiological Investigation and Case Identification

The LMPHU was notified of farmworkers who were symptomatic and presented to the pathology centre for SARS-CoV-2 viral PCR testing, which subsequently identified nine cases. Through contact tracing, further PCR testing, and rapid antigen testing, a cumulative total of 68 cases of the 74 farmworkers were identified, including 9 (13.8%) PCR-confirmed and 59 (86.8%) probable (Table 1). There were 42 staff contacts of which 3 later tested positive using a RAT and were able to isolate from their homes.

**Figure 1:**
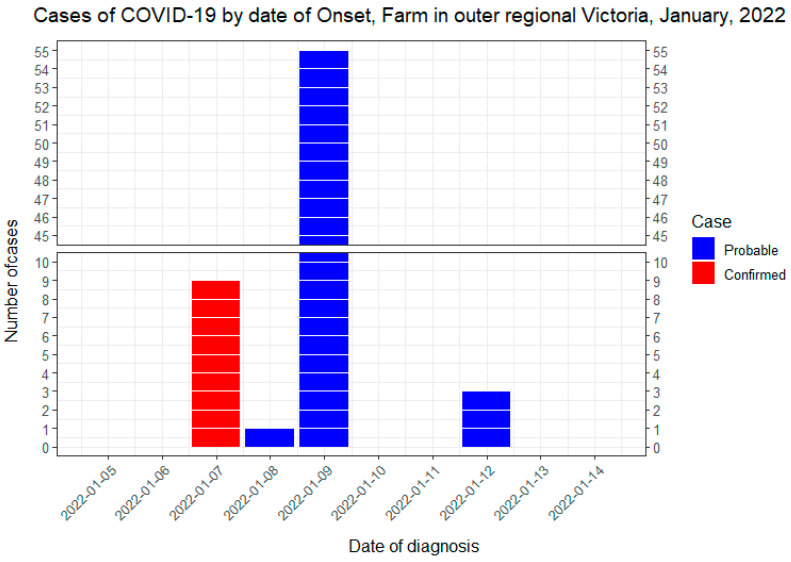
Epidemic curve of confirmed and probable cases. Nine cases were initially identified by PCR on 7 January 2022; then a further 56 showed positive RAT tests on 8 and 9 January, and the last 3 were identified on 12 January 2022.

Initially, three confirmed cases were notified to the LMPHU on 8 January 2022 with their samples collected on 7 January 2022. This result turnaround was considerably quick considering the national overload all pathology services were experiencing due to the peaking of the Omicron variant of concern (VoC) in Australia. All cases in this cohort were cleared by 19 January 2022 (Figure 1). Figure 1 shows this as a point source outbreak with the epi curve flattening after 9 January 2022 and the last three probable cases diagnosed on 12 January 2022. The SARS-CoV-2 virus VoC agent for this outbreak was not determined. All cases that were initially symptomatic on testing or during the infection period were assessed as asymptomatic at least 24 h before clearance date and released from isolation. COVID-19-negative contacts were tested on 9, 12, and 14 January 2022 and continued to quarantine until 19 January 2022 when the last positive cases were considered to be no longer infectious.

During the outbreak period, there were no deaths or requirements for hospitalisations among all workers; however, 10 (14.7%) cases had had minor COVID-19-related symptoms. Due to the few cases with minor symptoms, risk factors associated with the severity of infections could not be analysed. This cohort was mostly composed of male workers [57 (83.8%)]. The age group was mostly 30–39 years old [35 (51.5%)] followed by the 18–29 years old age group [24 (35.3%)]. All workers were younger than 50 years old (Table 1). Six workers that remained COVID-19-negative throughout the outbreak were all men with a median age of 28 years, whereas the case cohort had a median age of 32 years.

### 3.2. Outbreak Management and Implementation of Cohorting 

After the notification of initial cases on the 8 January 2022, the LMPHU declared the farm an outbreak site and initiated public health actions as per the public health and wellbeing act of 2008. An outbreak was defined as five or more cases in a high-risk residential setting in accordance with the DH COVID-19 control strategy. Identification of close contacts was completed, and requests for further testing and quarantine of all farmworkers were initiated, cohorting the positive cases after clinical assessment.

**Figure 2:**
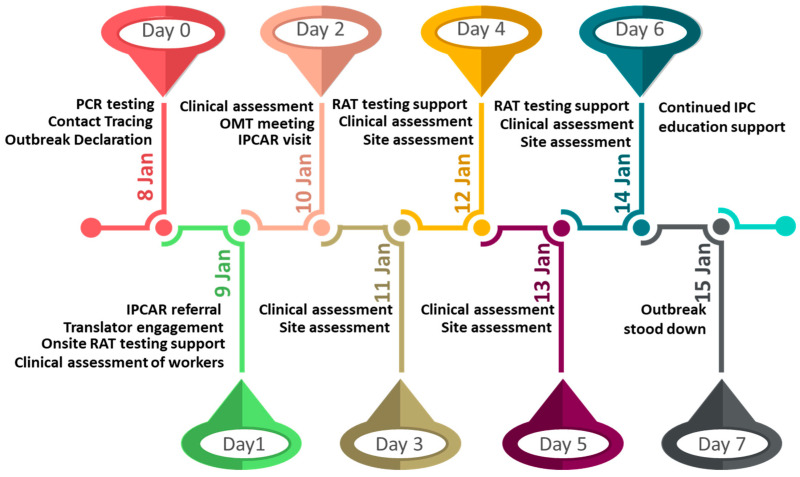
Timeline for key outbreak management interventions from date the outbreak was declared to date of stand-down.

On the second day of the outbreak, an IPCAR referral was made, and a site visit occurred on the third day (Figure 2). The visit highlighted the need for all staff to undergo training regarding the recommended infection control measures to prevent further spread of the SARS-CoV-2 virus. This visit also reinforced a two-step cleaning approach with a detergent to clean before using a disinfectant, the use of facemasks, and physical distancing. 

Cultural support and translation services were engaged on the second day as well and complimented the efforts for contact tracing, testing, and quarantine compliance as all workers had indicated Samoan as the preferred language. Further onsite RA testing and daily clinical assessment of all workers were performed before the outbreak was stood down on 15 January 2022 (Figure 2), noting that this was no longer a transmission site, i.e., all workers that did not test positive had completed their quarantine period.

All outbreaks managed by the LMPHU have an OMT. In this outbreak, an OMT was organised with farm management, the workers, and other stakeholders including pathology services, cultural and support services, an IPC consultant, medical leads, a local tertiary public hospital, and the local rural city council—emergency management. This OMT ensured that the farm was adequately supported to manage the outbreak; this was achieved through the provision of personal protective equipment, rapid antigen tests, and food for workers onsite. At the time of the OMT, more than 80% of the farmworkers were identified as either confirmed or probable cases. Considering the risks due to COVID-19 disease epidemiology, the LMPHU in liaison with the farm management provided an exemption for all asymptomatic individuals with positive RAT to be able to return to work with appropriate introduction of personal protective equipment (PPE). This was authorised by the deputy chief health officer and aided in ensuring that food distribution was not impacted. 

COVID-19 cases globally were mandated to stay at home and isolate. In workplaces, this minimised the spread of COVID-19 to workers who remained asymptomatic and tested negative, facilitating industry productivity and minimising impact on the economy as we transitioned to the “new normal” [14]. However, asymptomatic cases were able to choose to work from home whilst completing mandatory isolation, depending on their work requirements [15]. In this case study, after the initial screening, all cases were isolated, and, when more were identified, it was no longer possible to effectively isolate them but was much easier to quarantine their contacts. For this reason, all who remained negative could not return to work. The LMPHU recognised that it was important to ensure that asymptomatic RAT positive staff members were not coerced to attend work. As such, a cultural support officer external to the business regularly visited the site. This worker was able to communicate with cases in their preferred language and discussed with the cases their rights to ensure that they were not being coerced to attend work. The LPMHU director and medical lead advised that only cases who were asymptomatic and consenting were able to attend work. A further stipulation to this arrangement was that the farm health supervisor would be required to monitor staff to ensure that they were not presenting to work with symptoms. All cases received daily health monitoring through the local health service, and food relief was provided by the local multicultural support service. Both the farm health supervisor and cultural support worker provided language support to the cases; this was to ensure that instructions were communicated in preferred language for donning and doffing of personal protective equipment and for the use of rapid antigen tests. Overall, this public health response ensured improved health and social welfare of both cases and contacts throughout the outbreak period.

The positive farmworkers were cohorted in the accommodation onsite, whereas the negative workers were provided with single-room accommodation so that they could safely quarantine. All negative residents had to quarantine until all positive cases had completed their infection period, and effective cleaning of communal areas had been performed. This was ideal as there were challenges with fewer areas for appropriate quarantine, and only 6/74 (8.1%) workers had remained negative during the outbreak. Furthermore, the LMPHU engaged the IPCAR team to offer COVIDSafe training for workers in liaison with farm management on how to safely work with COVID-19-positive cases, PPE, isolation and quarantine requirements, and their implications. 

## 4. Discussion

With support from the LMPHU vaccination outreach team, many farms across the region were prepared for this pandemic and ensured that cases did not have severe outcomes. The outer regional and remote areas of Australia had virtually no COVID-19 in 2020 and the first half of 2021 due to government-imposed lockdowns. The rapid response teams involving the local pathology service, cultural and language support services, and farm management enabled adequate risk assessment and allowed for farm work to continue in the face of the outbreak. Even though the initiative to allow asymptomatic workers to continue working was not the norm for all outbreaks, it has also been reported elsewhere with favourable outcomes [16].

Although the viral genomics were not performed to identify the VoC in this outbreak, confirmation with PCR was important to understand the severity of the outbreak and negate the low sensitivity for the RAT as per DH guidelines [3]. Once established, follow-up tests for contacts were performed by RAT, and all who tested positive were considered probable cases. Although the outbreak coincided with the peaking of the Omicron VoC in the country, in this rural town, it is difficult to ascertain whether the causative agent was Delta or Omicron as infections have historically shown a delay compared with the more populated metropolitan regions. The high infectivity and low symptom severity may point towards a possible Omicron VoC as the causative agent [17,18,19]. 

In this case report, the farmworkers were managed by contractors and initially worked across different farms. Yet, they lived together in the farm’s accommodation camps where they shared facilities. This added another layer of complexity in contact-tracing and efficient communication between the farm management and contractors in identifying close contacts between different farms. However, after multiple site contact-tracing and testing, all contacts that lived elsewhere were able to quarantine in their homes, further reducing the risk of spread. No new cases or exposures were generated from these contacts. Other risks in this outbreak were mainly due to the timing coinciding with the peak of the Omicron COVID-19 VoC and the collapse of the pathology PCR testing capacity across the country. The RATs were introduced; however, the reporting system was not yet live online until the outbreak was well underway. Another challenge in managing this outbreak was that it occurred in a CALD group with low levels of written and spoken English. This proved a barrier to reading the provided instructions for responding to the outbreak, such as testing; reporting of RAT results; and compliance with isolation, quarantine, and appropriate facemask requirements. Furthermore, migrant populations have significantly higher proportions of undiagnosed co-morbidities and do not have access to many government support initiatives, such as Medicare, that promote equitable access to healthcare [20,21], which was reinforced during the COVID-19 pandemic [22].

Colignan is an outer regional town on the banks of the Murray River located at least 40 min from the rural city of Mildura where the main public hospital, testing, and vaccination clinics are located. Its remote location provides excessive challenges with access to healthcare. This cohort experienced barriers including the limited provision of appropriate local health options, limited transport, and fear of accessing services in many instances. The migrant farmworkers did not have Medicare cards, and whilst they did have private insurance, there were concerns expressed about the out-of-pocket costs they would incur should they need to access healthcare. Further communication barriers made it difficult to ensure that public health orders were complied with and testing requirements with RATs were correctly performed. As the case numbers were increasing during the outbreak, organising food and accommodation for effective isolation and quarantine became challenging.

Engagement with the farm management and cultural support for overcoming communication barriers was important for gaining trust between the LMPHU and workers who were living through a COVID-19 outbreak. The introduction and delivery of RAT kits further helped alleviate the challenges with PCR testing, which, at this point, had turnaround times not ideal to make useful public health actions. Although RATs are generally considered to have lower sensitivity to detect infections [23,24], they are regarded as a vital public health tool for the management of outbreaks [3]. The LMPHU has now engaged the services of an outer regional coordinator who will work hand-in-hand with key stakeholders such as community health services to specifically target more farms in the Mildura region for coordinated vaccination programs. 

### Lessons Learned

Outreach targeted testing and vaccination have allowed for the communities in the remote locations of the Loddon Mallee region to have access to vaccines, and this has subsequently reduced the burden of adverse events associated with COVID-19 infection. In our report, even though we had a large outbreak, the outcome of infection was excellent, the cases were still able to perform their work, and the productivity of the industry continued.

The trust between the LMPHU, farm management, pathology service, and cultural support made all the difference with the rapid response in screening, contact tracing, and innovative thinking and outbreak management to reduce spread and maintain workforce in this critical sector. The stakeholder engagement and cultural support including translation services for reporting test results were critical in evaluating the risk and extent of the outbreak. This provided the opportunity for the industry to continue to operate with a workforce that was asymptomatic yet RAT positive, and the few negative workers were able to safely quarantine, reducing the spread of infection whilst maintaining the workforce. The farm management and its workers readily embraced the COVIDSafe training for donning and doffing PPE allowing for public health actions to be implemented and complied with. Fostering existing relationships and building new ones with key stakeholders across the Loddon Mallee region was key to managing outbreaks. The collaborations have remained critical in gathering information, providing on-the-ground support, and achieving better outcomes for those impacted.

## 5. Conclusions

This farm outbreak in outer regional Victoria was the first major outbreak in the Loddon Mallee region during the time when the highly infectious Omicron VoC was spreading throughout Victoria. The pathology testing service was under unprecedented demand, and testing with RATs had just been introduced. The reporting system came online when this outbreak was underway. Furthermore, the outbreak occurred in a vulnerable population with limited English literacy, limited access to healthcare, and higher comorbidity per age compared with the rest of the country. This was a community of seasonal farmworkers who provide essential support to the agriculture industry, the mainstay of the economy in this part of the state. The LMPHU quickly acted to engage the farm management, local pathology service, and local health service and provided cultural and language support. This efficient collaboration enabled the continued productivity of the farm and provided extra training through the IPC consultant, which is and will be an invaluable asset for any future disease exposures and/or outbreaks.

## Figures and Tables

**Table 1:** Characteristics of COVID-19 cases in the outbreak (*n* = 68).

Characteristics of COVID-19 Cases	Number of Cases	Percent of Total
**Cases**		
Confirmed	9	13.8%
Probable	59	86.8%
**Clinical Assessment**		
No symptoms	58	85.3%
Symptomatic	10	14.7%
Hospitalisations	0	0.0%
Deaths	0	0.0%
**Sex**		
Men	57	83.8%
Women	11	16.2%
**Age Groups**		
18–29	24	35.3%
30–39	35	51.5%
40–49	9	13.2%

## Data Availability

Data available at reasonable request with permission from the Victorian Government Department of Health.
